# Best strategies to implement clinical pathways in an emergency department setting: study protocol for a cluster randomized controlled trial

**DOI:** 10.1186/1748-5908-8-55

**Published:** 2013-05-22

**Authors:** Mona Jabbour, Janet Curran, Shannon D Scott, Astrid Guttman, Thomas Rotter, Francine M Ducharme, M Diane Lougheed, M Louise McNaughton-Filion, Amanda Newton, Mark Shafir, Alison Paprica, Terry Klassen, Monica Taljaard, Jeremy Grimshaw, David W Johnson

**Affiliations:** 1 Division of Emergency Medicine, Children’s Hospital of Eastern Ontario, Ottawa, Canada; 2 Departments of Pediatrics and Emergency Medicine, University of Ottawa, Ottawa, Canada; 3 Children’s Hospital of Eastern Ontario Research Institute, Ottawa, Canada; 4 IWK Health Centre, Halifax, Canada, School of Nursing, Dalhousie University, Halifax, Canada; 5 University of Alberta, Edmonton, Canada; 6 Institute for Clinical Evaluative Sciences, Toronto, Canada; 7 Division of Paediatric Medicine, Hospital for Sick Children, Toronto, Canada; 8 Department of Paediatrics and Health Policy, Management and Evaluation, University of Toronto, Toronto, Canada; 9 College of Pharmacy and Nutrition, University of Saskatchewan, Saskatoon, Canada; 10 Departments of Pediatrics and of Social and Preventive Medicine, University of Montreal, Montreal, Canada; 11 Research Centre, CHU Sainte-Justine, Montreal, Canada; 12 Departments of Medicine (Respirology), Biomedical and Molecular Sciences (Physiology) and Community Health and Epidemiology, Queen’s University, Kingston, Canada; 13 ICES-Queen’s University, Kingston, Canada; 14 University of Ottawa, Ottawa, Canada; 15 Montfort Hospital, Ottawa, Canada; 16 Champlain Local Health Integrated Network, Ottawa, Canada; 17 Department of Pediatrics, Faculty of Medicine & Dentistry, University of Alberta, Edmonton, Canada; 18 Department of Emergency Medicine, Cambridge Memorial Hospital, Cambridge, Canada; 19 Michael G. DeGroote School of Medicine, McMaster University, Hamilton, Canada; 20 Ontario Ministry of Health and Long-Term Care, Toronto, Canada; 21 Faculty of Medicine, University of Manitoba, Winnipeg, Canada; 22 Manitoba Institute of Child Health, Winnipeg, Canada; 23 Department of Epidemiology and Community Medicine, University of Ottawa, Ottawa, Canada; 24 Ottawa Hospital Research Institute, Clinical Epidemiology Program, Ottawa, Canada; 25 Ottawa Hospital Research Institute, Ottawa, Canada; 26 Department of Medicine, University of Ottawa, Ottawa, Canada; 27 Division of Emergency Medicine, Alberta Children’s Hospital, Calgary, Canada; 28 Alberta Children’s Hospital Research Institute, Calgary, Canada; 29 Department of Pediatrics, Physiology and Pharmacology, University of Calgary, Calgary, Canada

**Keywords:** Clinical pathways, Key interventions, Intervention strategy, Pediatric emergency care, Theory-based strategy, Process outcomes, Clinical outcomes

## Abstract

**Background:**

The clinical pathway is a tool that operationalizes best evidence recommendations and clinical practice guidelines in an accessible format for ‘point of care’ management by multidisciplinary health teams in hospital settings. While high-quality, expert-developed clinical pathways have many potential benefits, their impact has been limited by variable implementation strategies and suboptimal research designs. Best strategies for implementing pathways into hospital settings remain unknown. This study will seek to develop and comprehensively evaluate best strategies for effective local implementation of externally developed expert clinical pathways.

**Design/methods:**

We will develop a theory-based and knowledge user-informed intervention strategy to implement two pediatric clinical pathways: asthma and gastroenteritis. Using a balanced incomplete block design, we will randomize 16 community emergency departments to receive the intervention for one clinical pathway and serve as control for the alternate clinical pathway, thus conducting two cluster randomized controlled trials to evaluate this implementation intervention. A minimization procedure will be used to randomize sites. Intervention sites will receive a tailored strategy to support full clinical pathway implementation. We will evaluate implementation strategy effectiveness through measurement of relevant process and clinical outcomes. The primary process outcome will be the presence of an appropriately completed clinical pathway on the chart for relevant patients. Primary clinical outcomes for each clinical pathway include the following: Asthma—the proportion of asthmatic patients treated appropriately with corticosteroids in the emergency department and at discharge; and Gastroenteritis—the proportion of relevant patients appropriately treated with oral rehydration therapy. Data sources include chart audits, administrative databases, environmental scans, and qualitative interviews. We will also conduct an overall process evaluation to assess the implementation strategy and an economic analysis to evaluate implementation costs and benefits.

**Discussion:**

This study will contribute to the body of evidence supporting effective strategies for clinical pathway implementation, and ultimately reducing the research to practice gaps by operationalizing best evidence care recommendations through effective use of clinical pathways.

**Trial registration:**

ClinicalTrials.gov: NCT01815710

## Background

The evidence to practice gap in medicine remains a healthcare challenge [1-8]. While knowledge syntheses and clinical practice guidelines (CPGs) have emerged as rigorous means to translate and make research more accessible for practitioners, these may not be sufficient to change practice behavior in complex settings, such as the chaotic environment of an emergency department (ED) [9,10] where there is also exceeding demand to achieve favorable wait times and patient throughput [11]. This pressure threatens the quality and safe care that are important to health providers who must contend with a diverse population of varying ages, medical conditions, and treatments. The clinical pathway (CP) has emerged as a potentially important knowledge translation strategy for promoting effective healthcare. As a clinical decision-making tool, CPs operationalize best evidence recommendations and CPGs into an accessible bedside format for health provider teams, and in this sense, can promote standardized evidence based practices, patient safety, and efficiency in the health system [11-21]. Well-designed CPs also offer opportunity to free clinicians’ cognitive abilities to focus on more complex thought-requiring activities [22] and can support clinicians to deliver key management priorities in a timely manner. As a result, CPs are being increasingly used in health settings and recommended by broader health systems internationally as a form of quality improvement [23,24].

While CPs have potential to link evidence to practice via integration of guidelines into local systems, and to improve patient outcomes while decreasing hospitalizations and other health costs, their true impact has been limited by variable implementation strategies and suboptimal research designs [25,26]. Because a CP involves the full health team and become part of the patient record, hospital contextual issues and team dynamics are important factors that must be considered in its implementation. Current evidence-based strategies that are used to implement CPGs may not be sufficient to promote CP adoption in hospital settings, because the complexities of behavior change among health providers are compounded by organizational and system barriers. Best strategies for implementing CPs are largely unknown [26,27] however, and this knowledge gap must be addressed before their full impact can be realized. Further study is needed to understand why and under which circumstances CPs lead to improved care [13,28,29].

Most CPs are developed internally within a hospital, and while contextual knowledge may facilitate local uptake, CP quality may be limited by lack of rigour and expertise locally in interpreting best evidence for incorporation into that pathway. Working at a broader level (e.g., provincial/state-wide, national) expert-developed CPs are created by multidisciplinary teams of clinical and research experts, including end users, and offer opportunity for high quality features and professional design. Expert CPs can also be a means to ensure the standard of care is provided across healthcare settings in different jurisdictions. Additional benefits include efficiencies in development and momentum for expert pathway updates with emergence of new evidence. However, expert CPs cannot simply be imposed and implementation at local levels can be challenging [30,31]. An effective intervention strategy requires thoughtful understanding of current and anticipated obstacles [32]. In this process, change management issues such as leadership, resources, and organizational culture must be explored and addressed.

This project seeks to provide new knowledge in an area of active ED practice and current interest. We will develop and evaluate the effectiveness of a theory-based intervention strategy using two expert-derived pediatric emergency CPs—Asthma and Vomiting and Diarrhea (V&D). These conditions have a strong evidence base for care, documented gaps in quality of care, and existing provincial leadership in pathway development [6,7,33-39]. Rigorous evaluation of key clinical and process outcomes related to each CP, and process evaluation of the implementation experience will identify important factors for implementation success that will be highly valuable in guiding future CP implementation strategies.

### Research objectives

We will conduct a 42-month mixed methods health services project with the following study objectives:

1. To design a theory-based and knowledge user-informed intervention strategy to implement two provincial pediatric emergency CPs into practice in community EDs.

2. To evaluate the effectiveness of this implementation strategy, using a cluster randomized controlled trial (cRCT) design, through measurement of relevant process and clinical outcomes in community EDs in Ontario, Canada.

3. To conduct a process evaluation of the implementation strategy.

4. To conduct an economic analysis of implementation costs and benefits.

## Methods

To study the effect of a theory based strategy on CP implementation, we will conduct two cRCTs to evaluate implementation of two different pediatric CPs, namely for asthma and V&D, in a sample of 16 Ontario community EDs. Developed by a multidisciplinary team of clinical and research experts, as well as end users, each CP was designed for broad dissemination for use in the emergency care of pediatric patients in any ED setting. To minimize any potential Hawthorne effect resulting from engagement alone, we will use a balanced incomplete block design [40]. We chose this design to ensure all sites are exposed to the intervention, thus balancing the Hawthorne effect. The design is ‘incomplete’ because the complete intervention will differ between the two groups. One-half of the ED sites will be allocated to the arm receiving the implementation intervention for the pediatric asthma CP (group one), while serving as a control for the Pediatric V&D CP intervention. The other half of the hospital sites (group two) will be allocated to the arm receiving the implementation intervention for the V&D CP, while serving as control for the asthma CP intervention. There will be no dissemination of documents or related materials to control sites. Using this design, we will be conducting two cRCTs while ensuring that all sites receive exposure to the intervention as we evaluate the implementation strategy for two different pathways.

### Sampling

#### Setting and site selection

A community ED will be defined as one that does not usually act as a referral center and is not a primary teaching hospital. Selecting from 149 community EDs in Ontario to ensure representativeness, we will enroll the 16 sites based on total patient volumes, namely very high (four sites), high (six sites), medium (four sites) and low (two sites).

#### Inclusion/exclusion criteria

We will recruit EDs that do not currently use CPs for either pediatric condition, asthma or V&D. To minimize contamination between sites, we will request data from Health Force Ontario, an organization that assists with physician coverage at different EDs, to ensure ED physicians at any site are not also working at a study site in the alternate arm of the study. An additional inclusion criterion is commitment to the implementation intervention by an administrative lead on behalf of the hospital. For each CP, specific inclusion/exclusion criteria have been defined for use with patients.

### Participants

The individual hospital sites and their ED teams will be the focus of this study. Participants at each site will include ED staff and physicians, as well as hospital administrators with responsibility for the ED. Clinical outcomes for pediatric patients (defined as <18 years of age) with either asthma or gastroenteritis will also be studied.

### Intervention strategy

Given the hospital context and involvement of the full ED team, CP implementation is a complex process requiring an informed and well-designed intervention strategy. To address the busy and competing pressures on EDs, an optimal intervention will be effective, practical, and feasible, yet with a strong evidence base for the design.

#### Core components

Our first objective is to fully develop the intervention strategy for optimal CP uptake at each site. Because our intervention strategy is dependent on ED site input, it is not possible to fully design the intervention until the study begins. However, we have provisionally selected the core intervention components with the collected knowledge gained from project team members’ experience with previous CP implementation and related initiatives [41-45]. These components address health professional, ED team and organizational (hospital) issues, and are summarized in Table 1. To ensure feasibility and sustainability, the implementation strategy will be designed for success within existing hospital resources. This is to minimize the impact of study support and infrastructure, and to assess implementation in a realistic setting without external support.

**Table 1:** Core components of implementation strategy

**Core component**	**Description**
Local site champion teams	Nurse educator or Emergency Nurse
	Emergency Physician
Pre-implementation site visits	Assessment of local ED culture, organization and feedback on CP usability (Human Design Factor Analysis)
Ongoing site support	Bimonthly teleconferences with site teams
Educational workshops	Train-the-trainer model
Website support	E-learning modules for each CP
	Resource materials
Posters/reminders	CP-specific visual tools
	Reminders to use CP on relevant charts
Hospital Commitment	Facilitation through hospital approval processes
	Allocation of hospital resources;
	Priorization within other hospital initiatives

#### Clinical pathway key interventions

Given their complex and interprofessional nature, we will identify each CP’s key interventions to ensure focus and emphasis on critical aspects for the pathway effectiveness. The development teams have identified evidence-based key interventions for each CP, and these will be the focus of training workshops, reminders and evaluations.

#### Theory-based design with knowledge user input

Our intervention strategy will be based on the Theoretical Domains Framework (TDF), which describes a comprehensive structure of 14 theoretical domains from 33 behavior change theories and 128 constructs [46]. This framework provides a useful approach to understand behavioral determinants and inform intervention design. A TDF-interview guide [47] is available to systematically elicit barriers and concerns from relevant stakeholders. To inform our strategy development, CP-specific behavioral domains will first be explored through TDF-guided [48] site visits (described below) and key informant (KI) interviews at each site. One administrator per site will be selected for KI interviews to identify readiness for change, as well as barriers and facilitators at the organizational level. Interviews will be audiotaped and transcribed. Data collection and analysis will follow an iterative and concurrent process [49].

#### Site visits

At the start of the implementation phase, site visits will be done to assess ED organizational issues, such as flow of pediatric patients, specific and shared roles of health providers, and current experience with CPs in the ED. Infrastructure requirements and readiness to implement the intervention CP will also be assessed. In previous implementation initiatives, this has proven to be very useful in understanding site issues and identification with the project. Additionally, site visits will be used to explore acceptability and potential issues with the intervention pathway. During each visit we will seek input from ED staff (*i.e.*, nurses and allied health professionals) and physicians on duty, using case scenarios to probe for clinical or procedurally important issues as we ‘walk through’ their intervention CP. A structured form will be used to guide these visits and capture field notes. To pilot this form and obtain preliminary information, we will do pre-site visits with two local EDs, exploring issues for one of the CPs in each. Site visit interviews will be audiotaped and transcribed for subsequent data analysis.

#### Intervention mapping

Information derived from the KI interviews and site visits will then be used to further develop site-specific intervention strategies. A mapping exercise will be conducted to link relevant behavioral elements identified to appropriate behavior change strategies [50]. We will apply known taxonomies of behavior change techniques [51] to identify relevant methods, and will select the optimal mode of delivery for each to create a multifaceted intervention strategy. To ensure implementation success, strong consideration will be held for feasibility and practicality. The full project team will be involved in the ultimate intervention design via web-conference meeting, and further input will be sought from site partners at our launch meeting.

#### Ongoing support and communication

We found from previous implementation experience that without frequent communication and support, the implementation becomes stalled and displaced by other priorities. Consequently, our strategy will include bimonthly teleconferences with site champions to discuss and support progress during the implementation phase, and every four months in the post-implementation phase to discuss sustainability issues. Teleconferences will be useful to share best practices and local solutions to address common barriers.

### Control sites

Control sites will continue with standard care, without additional materials. We will inform sites at recruitment that relevant pediatric patient data will be collected for ED presentations of asthma and V&D in the pre- and post-implementation periods. We will monitor relevant activities, such as self-implementation of any protocols that may relate to the control CP, within the process evaluation. While it is not possible to blind site partners to their allocated intervention, we will endeavor to conceal their allocation in the control arm. Control sites will not receive any CP documents. We will minimize potential co-interventions at recruitment by selecting only sites that do not have either related pediatric CPs in place. Based on recent studies of ED-based protocols [52,53], most community EDs in Ontario do not have an asthma or V&D CP in place.

### Randomization

Because the number of cluster sites is relatively small, simple unrestricted randomization would not be sufficient to ensure balance between the study arms. Pairing of hospitals would improve balance but may reduce study power and precision due to loss of degrees of freedom associated with a matched analysis. We will therefore implement a minimization procedure [54] to ensure overall balance based on three important covariates: annual pediatric visits, location (urban/rural), and recent experience with any ED process improvement initiatives (yes/no). A statistician not associated with the study will use a computerized algorithm to identify all possible allocations that meet the balancing constraints and one of the allocations will be randomly selected [55].

### Project phases

As shown in Table 2, the project is divided into five discrete phases. Following a nine-month preparation phase, the implementation phase will take place over the subsequent nine-month period. We have found that an open-ended implementation period is likely to lead to delays at various stages and might also complicate our data collection processes. For these reasons, we have designated a specific nine-month implementation period, with negotiated interim target dates, for all sites. Completed implementation will be defined when the following have been done: site-customization and committee approvals for the intervention CP, the CP is ready for use in the ED, delivery of at least two educational workshops, and promotion of the e-learning module through a central website. Our project coordinator will communicate regularly to ensure negotiated target deadlines are met. Should there be incomplete implementation at any site, reasons for this will be documented and explored further in the post-implementation interviews.

**Table 2:** Description of project phases

**Project phase**	**Duration**	**Activities**
Preparation Phase	9 Months	Site Recruitment & REB Approvals
		Site Randomization
		Intervention Development
		Project Launch Meeting
		Site Champion Training
Implementation Phase	9 Months	Site Readiness Visits
		Site Customization & Approval of Forms
		CP Training
Post-Implementation Phase	12 Months	Post-Implementation Site Visits
		Qualitative Interviews and Analysis
Data Collection and Analysis	7 Months	Chart Abstractor Training
		Chart Audits
		Chart to Administrative Database Linkage
		Quantitative Data Analysis
		Economic Analysis
Follow-up	5 Months	Full Partner Meeting: Review of Findings
		Wrap-up and Dissemination

### Evaluation

As shown in Table 3, we have planned a comprehensive evaluation approach including process and clinical outcomes. These are described in detail below.

**Table 3:** Evaluation components

**Quantitative evaluation**	**Qualitative evaluation**
- Patient Chart Audits	Process Evaluation
- Administrative Data	- Process Log
- Economic Analysis	- Pre/Post Site Visits
	- Pre-implementation Key Informant Interviews
	- Post-implementation Qualitative Interviews

#### Process evaluation

To address the third study objective, a concurrent process evaluation will be conducted throughout the trial to document and assess the degree of variability and fidelity in implementation of the intervention across the 16 sites. This evaluation will include a process log, and post-implementation site visits and qualitative interviews.

#### Process log

A process log will be used longitudinally to track key outcomes and capture issues related to site customization of documents, barriers and delays, workshop attendance and interest, ease of use, and degree of uptake. Components of this log will include workshop participant feedback and facilitator observation forms, brief bi-monthly feedback from site champion teams, and progress with negotiated target dates for various steps toward implementation. Additional support will be provided as needed to ensure targets are met. Website utilization, e-learning module feedback, and discussion boards will also be tracked to assess usefulness of the website as a complementary resource. We will use Google analytics (http://www.google.com/intl/en/analytics) to monitor website traffic and performance.

### Site visits

Using a standardized tracking form, site visits will be conducted two months after completed implementation to assess awareness, accessibility and ease of use of the CP. Findings at each site will be compared with pre-implementation site visits.

### Qualitative interviews

Post-implementation interviews will be conducted to gain a richer understanding of perceptions, team dynamics, and other relevant issues occurring at the individual and organizational levels. We will conduct post-implementation interviews at one-half of the sites for each intervention, for a total of 8 KI interviews and eight focus groups with up to ten ED provider participants each. Additional interviews will be conducted if data saturation is not achieved. To identify readiness for change and barriers and facilitators at the organizational level, one administrator per site will be selected for KI interviews. Interviews will be audiotaped and transcribed. At each focus group site, up to 10 ED health professionals with direct patient involvement will be invited to participate in a one-hour focus group session. The focus group moderator will record field note observations. A court reporter will do real-time transcription at all focus groups, a method that yields more complete and rapidly available records of greater fidelity [56]. Using the TDF as a coding framework, two coders will independently analyze all transcripts and field notes. Data collection and analysis will follow an iterative and concurrent process [49].

### Outcome measures

Given the nature of our study, both clinical and process outcomes are important to evaluate. Process outcomes relate to use of the CP for relevant patients. Clinical outcomes relate to adherence with key interventions as recommended by each pathway as well as patient specific outcomes. Table 4 presents a description of the specific outcome measures for this study.

**Table 4:** Process and clinical outcomes

**Outcome measure**	**Description**	**Details**
Primary Process Outcome	Completed CP on relevant patient charts	1) Initial: CP started; little or no documentation;
		2) Partial: some but incomplete documentation; or
		3) Full: meets requirements for CP success
Secondary Process Outcomes	CP use based on ED busyness	CP use for relevant patients, adjusted for shift-level ED data [66].
Primary Clinical Outcomes	Proportion of pediatric patients with asthma and V&D who received appropriate treatment, based on CP Key Interventions	Asthma CP: Treatment with corticosteroids [35,36,67,68] in the ED and at discharge, defined as:
		i). Patients with moderate to severe exacerbation are treated with systemic corticosteroids in the ED, and systemic plus inhaled corticosteroids at discharge,
		ii). Patients with mild exacerbation are treated with either inhaled or oral corticosteroids at discharge.
		V&D CP: Appropriate treatment with oral rehydration therapy (ORT), [37,39] defined as:
		i). Patients with moderate to severe dehydration are treated with ORT, and
		ii). Patients with no to mild dehydration are not treated with ORT.
Secondary Clinical Outcomes	Proportion of pediatric patients with asthma and V&D who received appropriate assessment or treatment, based on CP Key Interventions Patient specific outcomes	Asthma CP:
		Documentation of a Pre-school Respiratory Assessment Measure (PRAM) score [69-71].
		V&D CP:
		1) Documentation of a Gorelick score [37] for dehydration; and
		2) Proportion of children treated with intravenous therapy for rehydration [39].
		Both CPs: EDLOS, admission to hospital and re-visits to the ED within 72 hours.

Clinical outcomes will be measured through data abstracted from patient records and administrative databases in the 9-month pre and post-intervention periods. Prospective audits of the pre-implementation data are not possible as this period pre-dates the trial beginning. The post-implementation period can be clearly identified and prospective audit collection will therefore be done. Following completed implementation, we have scheduled a three-month settling in period before post-intervention audits will commence.

### Chart audits

Using a previously successful approach with community ED studies [57], we will ask local medical records departments to pull relevant charts using International Classification of Diseases-10 (ICD-10) codes for all diagnoses related to our index conditions (asthma and V&D) during the defined study periods. Chart auditors will review all records to ensure eligibility criteria are met. All retrieved and eligible patient charts will be audited. Based on data from previous work, and pediatric asthma visits in Ontario EDs [58], we estimate an average of 85 Asthma and 115 V&D patient charts per site during each nine-month pre- and post-intervention period. Therefore, at each site 200 charts will be audited in each period, for a study total of 6,400 chart audits. In the event of multiple visits for the same patient, data will only be collected for the index visit. Four health record auditors will be trained to abstract data from patient records and directly enter these into a secure online database. To assess inter-rater agreement, pairs of auditors will initially each abstract the same 100 charts (50 asthma, 50 V&D). Agreement will be measured with a kappa coefficient and further training will be done until a kappa >0.8 is achieved. A data dictionary will be created to guide the chart auditors and ensure standardized data collection procedures. Auditors will be blinded to the study aims, study design, and group allocation.

### Administrative data

Health administrative databases available at the Institute for Clinical Evaluative Sciences (ICES) will be used to study the impact of being on a CP on overall ED length of stay (LOS), hospital admissions, return visits within 72 hours following ED discharge for non-admitted patients, and death. Appropriate procedures will be used to link health administrative and patient specific data. The relevant databases are National Ambulatory Care Reporting System (NACRS), containing data from all Ontario EDs, and vital statistics data. Only data elements known to be reliable and valid will be used [59]. Use of NACRS data allows efficient capture of return visits, irrespective of whether the patient returns to the index hospital. These data also allow evaluation of shift-level characteristics, such as mean triage adjusted total LOS of all other patients in the ED on the same shift, which is an important contextual variable that may be associated with whether a CP was used and can be used to adjust for the LOS of the study patients. We will model whether the intervention had an impact on EDLOS, hospital admissions, return visits within 72 hours, and death; and whether ED busyness is associated with patients not being put on a pathway [66]. We will control for age, gender, Canadian Triage Acuity Scale (CTAS) rating, and EDLOS for other similar rated CTAS patients.

### Sample size calculation

We conducted sample size calculations for our primary clinical outcome measures using standard formulas for comparing two proportions in cluster-randomized trials [60]. We conducted separate calculations for each CP and selected the larger of the two requirements as our target sample size. We assumed an intracluster correlation coefficient (ICC) of 0.01. This was the largest ICC based on preliminary data from a similar, not yet published study, which showed ICCs for dichotomous outcomes ranging from 0.001 to 0.01. Based on preliminary data, we assumed proportions of 10% in the control arm. We anticipated average numbers of asthma and V&D patients per hospital over the nine-month study duration of 85 and 110 respectively. After applying 10% inflation to account for cluster size imbalances, and allowing for attrition of one hospital per arm, eight hospitals are required in each study arm to yield 90% power to detect an absolute difference of 10% between study arms (10% in the control arm versus 20% in the intervention arm) using a two-sided test at the 5% level of significance. At recruitment, hospitals will be asked for written commitment to the study. Regardless of whether full implementation at that site has been completed, all relevant patient charts will be audited as per the protocol described. Therefore, barring exceptional circumstances, it is unlikely that hospitals will be lost to follow-up.

### Analysis

#### Quantitative analysis

Corresponding to the incomplete block design, separate analyses, as described below, will be carried out for each CP. We will use descriptive statistics to compare hospitals and patient characteristics between the study arms: means and standard deviations will be calculated for continuous variables (or medians and interquartile ranges in the case of skewed distributions) and frequencies and proportions for categorical variables. Because we have a relatively small number of clusters per arm, the assumptions for mixed-effects logistic regression analyses and Generalized Estimating Equations (GEE) are not satisfied; primary analyses will therefore be carried out using cluster-level data [61].

We will first calculate hospital level summary measures (proportions in the case of dichotomous outcomes, or means in the case of continuous outcomes) in the pre- and post-intervention periods. A simple unweighted mean of the change from pre- to post-intervention will then be calculated for each hospital. The main effect measure for each outcome will be calculated as the difference between the mean changes in intervention and control arms, together with 95% confidence interval. If the distribution of the observed hospital summaries is markedly skewed, a logarithmic transformation will be applied, and the effect measures will be expressed as fold-changes. If descriptive analyses reveal important baseline differences in patient case mix between the study arms, a two-stage procedure will be used consisting of a standard regression analysis to obtain a residual adjusted for the covariates of interest in each hospital; hospital residuals will then be analyzed using the approach described above. Statistical significance will be assessed at the 5% level using a two-sample unpaired *t*-test.

We will conduct exploratory individual-level analyses using GEE to test whether ED busyness is associated with patients not being put on a pathway. Shift busyness will be measured as EDLOS for other similar rated Canadian Triage Acuity Scale (CTAS) patients. This analysis will also include the following individual-level covariates: patient age, condition (asthma, V&D) and CTAS rating.

#### Qualitative analysis

The focus groups and interviews will yield a substantial amount of complex data. To monitor progress and pursue emerging ideas, data collection and analysis will proceed concurrently [49]. The qualitative analyst will feed relevant emerging themes back to the focus group moderator and augment the interview guide as needed to capture any new ideas. Inductive analysis will be managed using N-VIVO software and will occur in three phases: coding, categorizing, and developing themes. As described earlier, all data will be coded using the TDF framework [46]. Codes will be broadly categorized corresponding to the major unit of analysis. As categories emerge, their theoretical properties will be defined. Comparisons between multiple categories will be carried out in order to locate similarities and differences between them. Finally, categories will be synthesized into themes. This process will be replicated for the qualitative data for each ‘case’.

#### Economic analysis

To address the fourth study objective, we will conduct an economic analysis [62-64] in parallel to assess costs associated with implementation versus benefits related to improvements in wait times, hospital admissions and ED revisits. To determine if the CP generates financial benefits (*i.e.*, savings), we will compare its use to standard care. We will also perform a decision analysis, from the hospital’s perspective with respect to costs, of implementation and uptake of both CP interventions. CP-associated costs will be divided into one-time start-up costs for implementation and recurrent costs for CP use and maintenance.

### Implementation costs

Throughout the project, we will track costs for all activities required for successful CP implementation at each site. This will include costs for: document customization and committee approval processes at each site, production of forms, posters and educational aides, preparation and delivery of workshops, and staff participation at these presentations. Additional costs to be tracked include refinement of web-based resources and access, travel and orientation for site champions and opinion leaders, telecommunication support, and other incidental activities. The hourly wage will be assumed to be the provincial mean for health professionals and administrators as per Ontario collective bargaining agreements, and $150 per hour for physician time.

#### Healthcare costs

Payer costs associated with emergency care for pediatric patients with asthma or V&D will be accounted for, based on ED visits, return visits, hospital admissions, and physician fees, which will be determined from the ICES administrative database. Costs for ED visits will be based on standard methodologies using resource intensity weightings (RIWs) available at ICES from the Canadian Institute for Health Information for hospital admissions and a derived RIW for ED visits. Physician fees associated with ED visits and admissions, including those for laboratory and diagnostic imaging services, will be captured through associated Ontario Health Insurance Plan billings and fee schedule. Cost data will be presented in Canadian dollars for the standard price year of 2012. To determine the effect of a full CP implementation, these health costs will also be compared for all patients at each site presenting with asthma and V&D in the pre- versus post-implementation phases.

### Synthesizing costs and effects

In the final economic evaluations, additional costs and outcomes will be synthesized in an incremental cost-effectiveness ratio (ICER) comparing the intervention versus control. This will result in two cost-effectiveness analyses (CEAs) relating to each CPs. The ICER for each CEA will be expressed as the incremental costs per proportion of change related to the primary clinical outcome for that CP.

### Uncertainty analysis

In the final analysis, because an economic evaluation is always surrounded with uncertainty [64] the robustness of the ICER will be checked for sample uncertainty using non-parametric bootstrapping. The bootstrapped cost-effectiveness ratios will be subsequently plotted in a cost-effectiveness plane, in which the vertical line reflects the difference in costs and the horizontal line reflects the difference in effectiveness. The choice of treatment depends on the maximum cost, known as the ceiling ratio, that society is prepared to pay for a gain in effectiveness. Therefore, the bootstrapped ICERs will also be depicted in a cost-effectiveness acceptability curve [65] showing the probability that the intervention is cost-effective using a range of ceiling ratios.

### Ethics and registration

Ethical approval has been granted for this study at the coordinating hospital (Children’s hospital of Eastern Ontario Research Ethics Board), and we are seeking site ethics approval at each hospital as their study participation is confirmed. Informed consent will be sought for interviews and focus group meetings. All research data will be stored on a secure server and will be transmitted through a secure virtual private network (VPN). It will then be securely deleted from the laptop hardrive using the software Eraser (http://www.eraser.heidi.ie). This trial is registered with ClinicalTrials.gov (http://www.clinicaltrials.gov/NCT01815710).

### Trial status

We are currently in the preparation phase, enrolling sites and seeking local ethics approvals.

## Discussion

CPs represent an important opportunity to narrow the evidence to practice gap in specific clinical settings. Given their interprofessional and typically hospital-based nature, integration of CPs into these settings involves complex interventions. Further evidence is needed, however, to guide effective methods for successful CP implementation. We have described a comprehensive study that will generate new knowledge based on a theory-based intervention strategy to implement CPs in community hospital settings. This study will address behavior change among health professionals, interprofessional team issues, patient outcomes and health economic impact. While CP outcomes are of ultimate importance, they cannot be viewed as proxies for implementation success. Through evaluation of a broad set of process and clinical outcomes, patient outcomes and clinician management—based on key interventions for each CP—can be assessed relative to degree of success in achieving implementation process measures. This knowledge can then be leveraged to inform future CP implementation activities.

We expect our study findings to be generalizable to other settings. By including EDs with varying sizes, urban/rural locations and target population patient visits, we will gain a richer understanding of relevant implementation issues across different situations. Through site visits, interviews, and continued communication throughout implementation, we also hope to identify important considerations that can be generalized to other CPs. To ensure a pragmatic approach, we have planned intervention components that can be achieved without significant additional workload or resources. Less reliance on study support and infrastructure will provide insight into success of future implementations without any such external support.

An obvious challenge for this project relates to the complex nature of the interventions being studied, and our potentially limited control in some aspects of implementation. However this affirms the importance of conducting this work in a research capacity, with a rigorous concurrent process evaluation. Another challenge relates to ED and hospital issues in particular. EDs are chaotic settings with multiple and often varying health professional team members. By focusing on pediatric conditions, which are typically an identified need in community EDs, we may uncover general issues with pediatric emergency care in these settings; many of these cannot be addressed with our intervention. Other hospital issues, such as competing priorities, budget restrictions, and staff turnover may also challenge implementation success at some of our sites. However, these are real issues confronting hospitals and must be considered for any implementation strategy.

Our research findings will be important to health professionals and organizations that are impelled to deliver evidence-based care. We will contribute to the body of evidence supporting effective strategies for CP implementation, and ultimately reducing the research to practice gaps by operationalizing best evidence care recommendations through use of CPs.

## Abbreviations

CEAs: Cost-Effectiveness Analyses; CPs: Clinical Pathways; CPGs: Clinical Practice Guidelines; cRCT: Cluster Randomized Controlled Trial; CTAS: Canadian Triage Acuity Scale; ED: Emergency Department; GEE: Generalized Estimating Equation; ICC: Intracluster Correlation Coefficient; ICD-10: International Statistical Classification of Diseases; ICER: Incremental Cost-Effectiveness Ratio; ICES: Institute for Clinical Evaluative Sciences; KI: Key Informant; LOS: Length of Stay; NACRS: National Ambulatory Care Reporting System; RIWs: Resource Intensity Weightings; TDF: Theoretical Domains Framework; V&D: Vomiting and Diarrhea; VPN: Virtual Private Network

## Competing interests

The authors declare that they have no competing interests.

## Authors’ contributions

All authors contributed to the design of this study protocol. Much of this planning process began during a planning and development meeting (funded by a CIHR Meetings, Planning and Development grant) and continued during teleconference or email communications. MJ developed the manuscript draft. All authors read and approved the final manuscript.

## Authors’ information

The team includes expertise in knowledge translation (JG, DJ, JC, SDS, FD), clinical pathways (MJ, DJ, MDL, FD, SDS, TR), trial methodology (DJ, TPK, MDL), biostatistics (MT), health service research (AG), health economics (TR), ED administration (MJ, LMF, MS) and pediatric emergency care (MJ, DJ, TPK, FD). AP provides expertise in health policy and health system strategy, and will ensure that the findings from the study are disseminated through the Ontario Ministry of Health and Long-Term Care.
